# Study of the mass balance, biotransformation, and safety of [^14^C]IBI351 in healthy Chinese subjects

**DOI:** 10.7555/JBR.38.20240254

**Published:** 2024-10-22

**Authors:** Shuaishuai Wang, Wen Lin, Bilal Ahmed, Tianqi Zhong, Jun Zhao, Lijun Xie, Hao Feng, Juan Chen, Chen Zhang, Peng Yan, Shirui Zheng, Lingge Cheng, Yipeng Cheng, Bei Zhu, Feng Han, Lulu Zhang, Chen Zhou

**Affiliations:** 1 Medical Basic Research Innovation Center for Cardiovascular and Cerebrovascular Diseases, Ministry of Education, International Joint Laboratory for Drug Target of Critical Illnesses, Key Laboratory of Cardiovascular and Cerebrovascular Medicine, School of Pharmacy, Nanjing Medical University, Nanjing, Jiangsu 211166, China; 2 Phase Ⅰ Clinical Trial Unit, the First Affiliated Hospital of Nanjing Medical University, Nanjing, Jiangsu 210029, China; 3 Value Pharmaceutical Services Co., Ltd., Nanjing, Jiangsu 211806, China; 4 Nuclear Medicine Department, the First Affiliated Hospital of Nanjing Medical University, Nanjing, Jiangsu 210029, China; 5 Innovent Biologics Co., Ltd., Shanghai 201107, China

**Keywords:** KRAS G12C inhibitor, IBI351, pharmacokinetics, metabolism, mass balance, radiolabel study

## Abstract

IBI351, a synthetic compound, exerts its anti-tumor effects by specifically, covalently, and irreversibly modifying the 12th cysteine residue of KRAS G12C. However, the pharmacokinetic profile of IBI351 in humans has not yet been reported. The current study aimed to investigate the pharmacokinetics and safety of IBI351 in healthy Chinese male subjects. A single oral dose of 600 mg combined with 150 μCi [^14^C]IBI351 was administered to six healthy male volunteers. Blood, urine, and fecal samples were collected at multiple time points to quantify the parent drug and its metabolites. IBI351 showed favorable pharmacokinetic characteristics and was well tolerated by all participants. Seventeen major metabolites were identified in plasma, urine, and feces. The main metabolic pathways included oxidation, hydrogenation, sulfonate conjugation, glucuronide conjugation, and cysteine conjugation. Excretion of IBI351 and its metabolites occurred mainly through feces. Collectively, this first-in-human study provides essential data on the metabolism and safety of IBI351 in Chinese subjects and lays the foundation for its further clinical development as a novel anti-tumor drug.

## Introduction

Rat sarcoma virus (*RAS*) genes comprise three proto-oncogenes: *HRAS*, *KRAS*, and *NRAS*. Among these, mutations in *KRAS *are the most common genetic alterations observed in lung adenocarcinoma, pancreatic cancer, and colorectal cancer^[1–2]^.

The *KRAS* gene encodes a 21 kDa small GTPase protein primarily localized to the intracellular surface of the cell membrane. KRAS functions as a downstream effector of the epidermal growth factor receptor family and regulates cellular growth and proliferation through complex regulatory mechanisms^[3–4]^. KRAS is inactive after binding to guanosine diphosphate (GDP) but active upon binding to guanosine triphosphate (GTP). In various cancers, KRAS is abnormally activated when stimulated by certain signaling factors, thus activating downstream signaling pathways, including the mitogen-activated protein kinase pathway, the phosphoinositide 3-kinase signaling pathway, the Ral guanine nucleotide dissociation stimulator-Ral signaling pathway, and other signaling pathways that promote cell survival and proliferation^[5]^.

*KRAS* mutations predominantly occur at codons 12, 13, and 61, most commonly at codon 12. Specifically, the G12C mutant is frequently observed in non-small cell lung cancer and colorectal cancer. In this mutant, the glycine residue at position 12 of KRAS is substituted with cysteine^[6–7]^. The KRAS G12C mutation disrupts the normal balance between GDP and GTP binding, reduces the binding of KRAS to GTPase-activating proteins (GAPs), and leads to an overactivation of KRAS in its GTP-bound state, ultimately promoting tumor initiation and growth. Additionally, *KRAS* mutations remodel the tumor microenvironment by inhibiting anti-tumor immune responses, further promoting tumor progression^[8]^. Notably, Shokat and colleagues have discovered that the allosteric pocket near the cysteine residue in the KRAS G12C mutant (switch Ⅱ pocket) may be targeted by small molecules to lock the mutant KRAS in an "inactive" state without affecting the wild-type KRAS protein, inspiring the design of inhibitors that directly target KRAS^[9–10]^. Two KRAS inhibitors, sotorasib (AMG-510, Amgen, Thousand Oaks, CA, USA) and adagrasib (Mirati, San Diego, CA, USA), have been approved for clinical use and have demonstrated high efficacy and safety^[11–12]^. Additionally, other KRAS G12C covalent inhibitors, including divarasib (Genentech, South San Francisco, CA, USA) and MK-1084 (Merck, Kenilworth, NJ, USA), are undergoing clinical trials^[13–15]^.

IBI351, with a chemical formula of C_32_H_30_ClFN_6_O_4_, is a synthetic compound developed by Innovent. It acts by specifically, covalently, and irreversibly modifying the 12th cysteine residue of KRAS G12C^[16]^. This modification effectively inhibits the GTP/GDP exchange on KRAS G12C, thereby locking KRAS in an inactive GDP-bound state and reducing the levels of GTP-bound activated KRAS. IBI351 exerts its anti-tumor effects by directly inhibiting the mitogen-activated protein kinase signaling pathway through downregulation of KRAS^[16]^. This leads to cell cycle arrest at the G1 phase, thereby inhibiting tumor cell proliferation. Additionally, IBI351 induces apoptosis in tumor cells by repressing other downstream signaling pathways^[16]^.

To date, the metabolism, excretion, and mass balance characteristics of IBI351 in humans remain unclear. Therefore, we conducted a study involving six healthy Chinese subjects to investigate the distribution, elimination, and metabolism of IBI351 after a single oral dose labeled with ^14^C.

## Subjects and methods

### Research design

**Figure 1:**
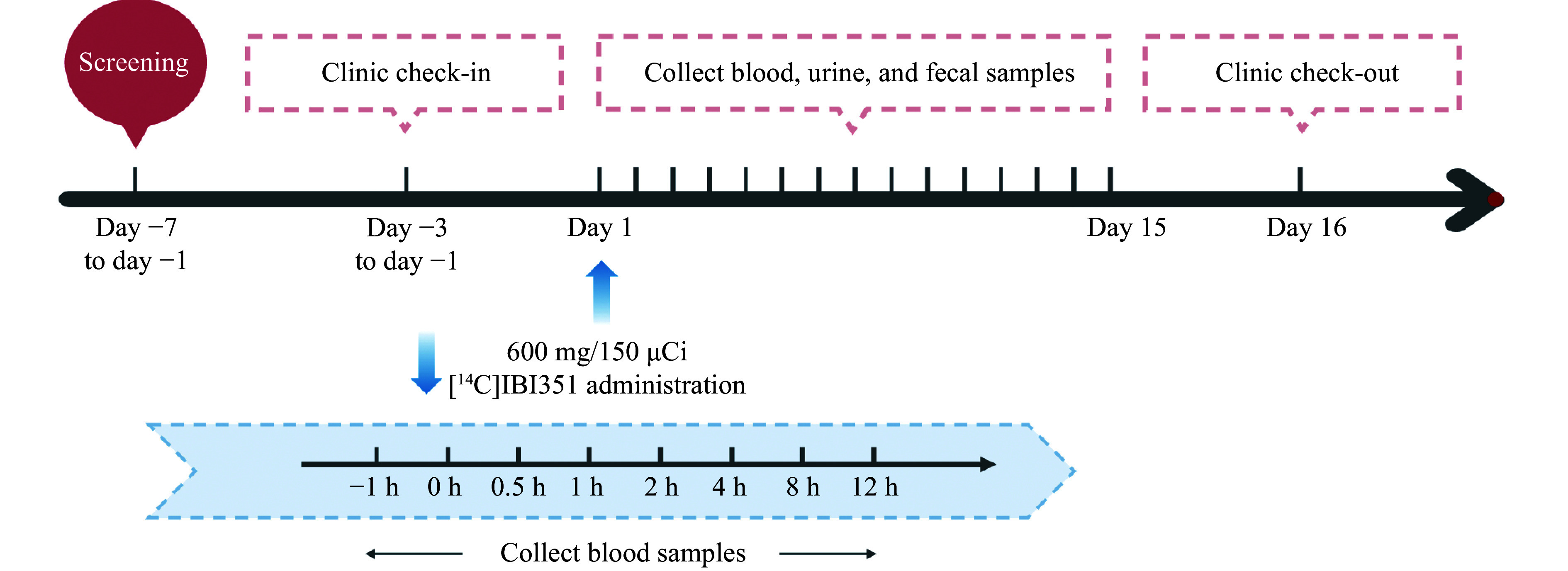
The experimental flow.

This was a single-center, single-dose, open-trial study involving six healthy Chinese male subjects. They received a single dose of 600 mg/150 μCi [^14^C]IBI351 on day 1 after fasting for at least 10 h. Blood, urine, and fecal samples were collected at specified time points or intervals within 15 days (0–336 h) following the administration of [^14^C]IBI351. The experimental procedure flow is shown in ***Fig. 1***.

The current study aimed to analyze the pharmacokinetic parameters of [^14^C]IBI351 and the ratio of total radioactivity (TRA) in the whole blood and plasma. The mass balance data were obtained, and the main excretion pathways in humans were identified by quantifying the TRA in the excreted samples from healthy subjects after [^14^C]IBI351 administration. Moreover, the main metabolites in the plasma, urine, and feces were analyzed to characterize the radioactive metabolite profile. The safety of [^14^C]IBI351 was also evaluated. The current study was reviewed and approved by the Drug Clinical Trial Ethics Committee of the First Affiliated Hospital of Nanjing Medical University, and all subjects were fully informed and signed an informed consent (ethical approval number, 2022-MD-214; drug clinical trial registration number, NCT05626179). The study was conducted in accordance with the local legislation and institutional requirements.

The experimental IBI351 raw material was provided by Innovent Biologics Co., Ltd (Suzhou, China). The [^14^C]IBI351 oral formulation was manufactured by Value Pharmaceutical Services Co., Ltd (Nasr City, Cairo, Egypt).

### Participant selection

According to the FDA guidance document entitled "Clinical Pharmacology Considerations for Human Radiolabeled Mass Balance Studies" (Docket Number: FDA-2022-D-0113), six healthy Chinese male subjects aged 18–49 years with a body mass index of 19–26 kg/m^2^ were eligible. Subjects were excluded if they met any of the following exclusion criteria: (1) a history of drug allergy, donation of blood, or significant blood loss (> 200 mL) within three months before the trial, or received a blood transfusion within one month; (2) a history of cardiac, respiratory, endocrine, renal, gastrointestinal, skin, infectious, hematological, neurological, or psychiatric diseases; (3) conditions including chronic constipation or diarrhea, irritable bowel syndrome, inflammatory bowel disease, and/or perianal disease; (4) factors affecting drug absorption, distribution, metabolism, and/or excretion; and (5) previous participation in radiolabeled clinical trials or exposure to significant radiation (*e.g.*, more than one session of X-ray, computed tomography scan, or barium meal, and involvement in radiation-related occupations) within the past 12 months.

### Drug dose, preparation, and administration

The recommended dose of 600 mg was suggested for the phase Ⅱ clinical trial of [^14^C]IBI351, based on the safety and efficacy demonstrated in a phase Ⅰ clinical trial in patients with advanced solid tumors harboring the KRAS G12C mutation. Additionally, considering the previous clinical exposure to the drug and the sensitivity in detecting and analyzing radioactive plasma samples, the oral dose of the drug in the trial was set at 600 mg/150 µCi [^14^C]IBI351. The radiopharmaceutical dose of 150 µCi used in this trial fell within the minor-risk category for human radiation exposure. Based on our preliminary calculations, the theoretical radiation doses received by adult male subjects were approximately 0.0101 mSv for bone marrow, 0.00276 mSv for testes, and negligible for the lens. The effective dose to the whole body was 0.0972 mSv. These values were well below the radiation dose limits specified by the Food and Drug Administration in 21 CFR 361.1 (30 mSv) and complied with the International Commission on Radiological Protection standards for radiation dose limits, indicating a risk level considered negligible, classified as Category Ⅰ.

The [^14^C]IBI351 oral formulation was reconstituted as a suspension using drinking water for oral administration to fasting subjects in the morning, ensuring complete consumption within 10 min. The total volume administered, including medication and water, was approximately 240 mL.

### Sample collection and processing

The endpoint of blood analysis was a plasma radioactivity concentration less than 1/8 of the baseline plasma value. Venous blood samples were collected from the six subjects (01101–01106) at the following time points (in hours): −1 (pre-dosing), 0.5, 1, 2, 4, 8, 12, 24, and at 24-h intervals (post-dosing). Whole blood aliquots were collected for radioactivity measurement, and blood samples were centrifuged (1500 [± 10] *g*, 4 ℃, 10 min) for metabolite identification. All samples were stored at −10–−30 ℃ until analysis.

Urine and fecal samples were collected for mass balance analysis and metabolite profiling. Urine samples were collected in polyethylene containers during the following time periods (in hours): −24–0 (pre-dosing), 0–4, 4–8, 8–12, 12–24, and at 24-h intervals thereafter (post-dosing) and stored at 4 ℃ during the collection period. Fecal samples were collected during the following periods (in hours): −24–0 (pre-dosing), 0–24, and at 24-h intervals thereafter (post-dosing). The collection of urine and fecal samples was terminated when the TRA in the combined biological samples (urine + feces) from each subject exceeded 90% of the administered dose. Additionally, the radioactivity in samples collected over two consecutive days should be less than 1% of the administered dose. The samples were weighed in special containers and stored at −10 ℃ to −30 ℃ until analysis.

Samples of the whole blood, plasma, urine, and feces were collected from these six healthy Chinese male subjects (01101–01106) as follows: whole blood samples were collected before and within 48 h after drug administration (seven time points for each case). Plasma samples were collected before and within 456 h after drug administration for subjects 01101 and 01103 (22 time points for each case); before and within 384 h after drug administration for subject 01102 (21 time points); and before and within 408 h after drug administration for subjects 01104 and 01106 (19 time points for each case), and 01105 (14 time points). Urine samples were collected before and within 312 h after drug administration for all subjects (01101–01104 and 01106: 17 time points for each case; 01105: 14 time points). Fecal samples were collected before and within 312 h after drug administration for subjects 01101–01103 (14 time points for each case); before and within 288 h after drug administration for subject 01104 (10 time points); before and within 240 h after drug administration for subject 01105 (11 time points); and before and within 288 h after drug administration for subject 01106 (12 time points).

### Radioactivity analysis

Blood, plasma, urine, and fecal samples were analyzed for TRA using the Tri-Carb model 3110 TR liquid scintillation counter (PerkinElmer, Waltham, MA, USA). Blood samples were divided into two parallel portions (0.5 g) and completely burned by OX-501 oxidizer (R.J. Harvey, Tappan, NY, USA). Finally, the samples were analyzed by liquid scintillation counting. Plasma (0.5 g) and urine (1 g) samples were directly analyzed by liquid scintillation counting. Fecal samples were first homogenized with isopropanol/water (50/50, v/v). The excretion of radioactivity in the urine and feces, referring to the cumulative recovery of total radioactive material in each time period, was defined as the sum of the percentage of TRA in the urine and feces relative to the administered dose. The TRA in each sample during a time period and the percentage of TRA related to the administered dose (% of dose) were calculated using the following formula: % of dosage = (TRA detected in the sample/radioactive dosage) × 100%.

The radioactivity in the whole blood and plasma is represented by the TRA ratio between the whole blood and plasma.

### Quantification analysis of IBI351

Plasma, urine, and fecal samples were analyzed using high-performance liquid chromatography-tandem mass spectrometry (HPLC-MS/MS; ABSciex, Carlsbad, CA, USA) to determine the concentrations of IBI351. Samples containing stable isotope-labeled GF1927 (C_32_H_27_D_3_ClFN_6_O_4_, with a molecular weight of 620.10) as an internal standard were subjected to protein precipitation with acetonitrile and separated on a Waters Xbridge C18 column (3.5 μm, 50 mm × 2.1 mm; Waters, Milford, MA, USA) at 40 ℃. The mobile phase consisted of deionized water (with 0.1% formic acid) and acetonitrile (with 0.1% formic acid), delivered by gradient elution at 0.6 mL/min, and the gradient elution program was 30% phase B in 0–0.29 min, 80% phase B in 0.30–1.80 min, 30% phase B in 1.81–3.00 min. The detection was performed on a Triple Quad 6500+ tandem mass spectrometer in the positive ion electrospray ionization mode. Quantification was conducted by multiple reaction monitoring of the transitions m/z 617.3→358.2 for IBI351 and m/z 622.2→363.1 for internal standard, respectively. The calibration range for IBI351 in human plasma was 5–5000 ng/mL.

### Metabolite detection and analysis

A plasma sample (0–144 h) was obtained by pooling plasma at each time point in a volume proportional to the time interval used for calculating the area under the concentration-time curve (AUC) (AUC_0–144 h_ pool) for each subject. Moreover, four plasma samples at 1 h, 4 h, 48 h, and 96 h were obtained by mixing them in equal volumes. Urine samples were pooled across individuals at an equal volume to obtain one pooled sample within each time period of 0–24 h, 24–48 h, and 48–96 h. Fecal samples (0–192 h) were pooled at the same weight percentages across collection intervals for each subject. Moreover, fecal samples were pooled across individuals at an equal weight to obtain one pooled sample within each time period of 0–72 h, 72–120 h, and 120–192 h. Plasma, urine, and fecal homogenates were extracted and concentrated using organic solvents, and then the redissolved solutions were used for metabolite profile analysis. The plasma, urine, and fecal extracts were separated on HPLC columns, and the HPLC flow fractions were collected in Deepwell LumaPlateTM-96 plates at 15 s/fraction using an automatic fraction collector. The radioactivity of each fraction was quantified using a microplate detector with ARC^®^ Convert and Evaluation software (3.0.2.379) and transformed to obtain the radioactive metabolite spectra. The radioactive metabolite spectra of each sample were integrated to acquire the peak area of each radioactive metabolite peak, the proportion of each spectral peak to the total radioactive intensity of the sample, and the proportion of the radioactivity of the major metabolites to the TRA in the corresponding blood, urine, and fecal samples. Therefore, the ratio of metabolites' concentrations to the total drug exposure (AUC) in the plasma and the ratio of each metabolite's level to the dose administered in the urine and feces were calculated. Liquid chromatography with low-energy radionuclide detection and mass spectrometry was used to identify the main metabolites in human plasma, urine, and fecal samples and to identify the possible biotransformation pathways of IBI351 in these samples.

### Pharmacokinetics analysis

The plasma levels of TRA and the plasma concentration of IBI351 were analyzed to determine pharmacokinetic parameters, including maximum concentration (*C*_max_), time to reach *C*_max_ (*T*_max_), area under the curve from time zero to one hour (AUC_0–1h_), area under the curve from time zero to the last quantifiable concentration (AUC_0–last_), area under the curve from time zero to infinity (AUC_0–inf_), elimination half-life (*T*_1/2_), apparent distribution volume corrected by bioavailability (*V*_z_/*F*), and apparent clearance rate corrected by bioavailability (*CL*/*F*). In the calculation of *C*_max_ and AUC of TRA, one gram of the plasma was considered equivalent to 1 mL.

### Safety analysis

Safety was evaluated based on the adverse events that occurred during the trial. Normal/abnormal changes in physical examination, vital signs, laboratory tests, electrocardiogram, and other results were evaluated before and after the trial.

### Drug exposure analysis

The exposure level of [^14^C]IBI351, including information on the actual dose of the drug, was measured to analyze drug exposure. The actual dose of IBI351 (mg) was calculated based on the specific activity and the actual radioactive dose consumed.

### Statistical analysis

Statistical analysis of TRA in the whole blood and plasma, concentrations of IBI351, corresponding pharmacokinetic parameters, and urine and fecal excretion data was performed using Microsoft Excel 2010. The pharmacokinetic parameters were calculated using Pkanalix 2021R1 (Lixoft, Antony, France). The drug-time curve was plotted using WinNonlin® (version 8.3, Certara). Data were analyzed using the SAS 9.4 software and presented as mean ± standard deviation.

## Results

### Demographics and other baseline characteristics

All the six male subjects were Han Chinese aged 23–36 years, with a mean age of 30.2 (± 4.54) years, a mean weight of 69.9 (± 5.54) kg, and a mean body mass index of 22.9 (± 1.29) kg/m^2^. ***Supplementary Table 1*** (available online) provides a summary of the key demographic characteristics. All enrolled subjects underwent baseline physical examinations, vital sign assessments, routine laboratory tests (including complete blood count, blood biochemistry, urinalysis, and coagulation function), chest X-ray, and 12-lead electrocardiogram examination. No significant abnormalities were observed during these assessments, and only some clinically irrelevant changes (such as diarrhea, elevated blood triglycerides, and positive occult blood in urine) were detected within the normal range.

### Drug administration

All the six subjects received the planned single oral dose of 600 mg/150 μCi [^14^C]IBI351 suspension. The average actual administered dose of IBI351 was 591 mg, and the actual radiation dose ingested was 146 μCi. The actual ingested radiation dose of [^14^C]IBI351 was determined by subtracting the residual radiation dose in the container after administration from the total radioactive dose in each formulation. The data are summarized in ***Supplementary Table 2*** (available online).

### Mass balance and excretion

**Figure 2:**
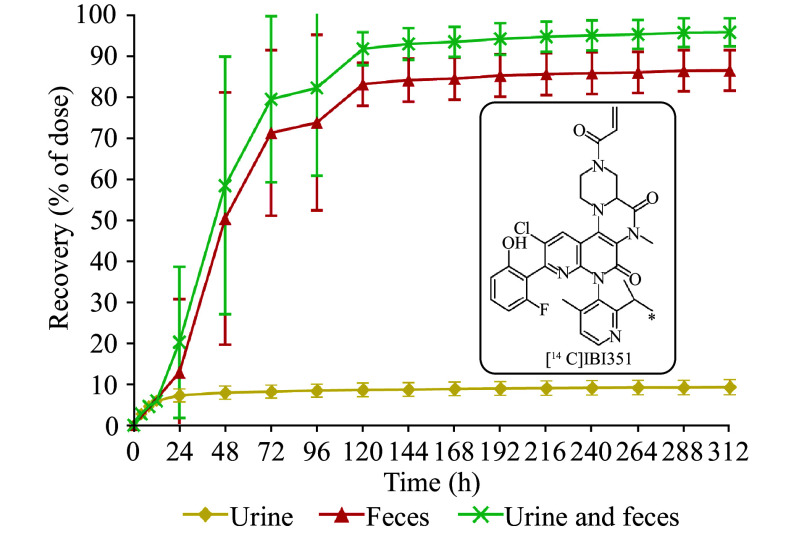
Cumulative excretion ratios in the urine and feces. Data are represented as mean ± standard deviation after a single oral dose of 600 mg/150 μCi [^14^C]IBI351 (*n* = 6). The asterisk (*) indicates the position of ^14^C-labeling.

After a single oral dose of 600 mg/150 μCi of [^14^C]IBI351 in the six subjects, the total radioactive recovery rates from urine and feces are shown in ***Supplementary Table 3*** (available online), and the cumulative percentage recovery rate is shown in ***Fig. 2***.

The percentage of total radioactive excretion (% of dose) in the urine and feces was 95.56% (± 3.41%) after administration (0–312 h), with the majority (86.30% [± 4.90%]) of the radioactive content excreted in feces and a small amount (9.27% [± 1.83%]) excreted in urine. Total radioactive content was primarily (more than 90.00% on average) excreted in urine and feces within 120 h (five days) after administration. At 144 h (six days) post-dose, the excretion rate decreased to less than 1.00%. The renal clearance of IBI351 was 1.82 L/h.

### Pharmacokinetic parameters

#### TRA in the plasma and the main pharmacokinetic parameters of IBI351

**Figure 3:**
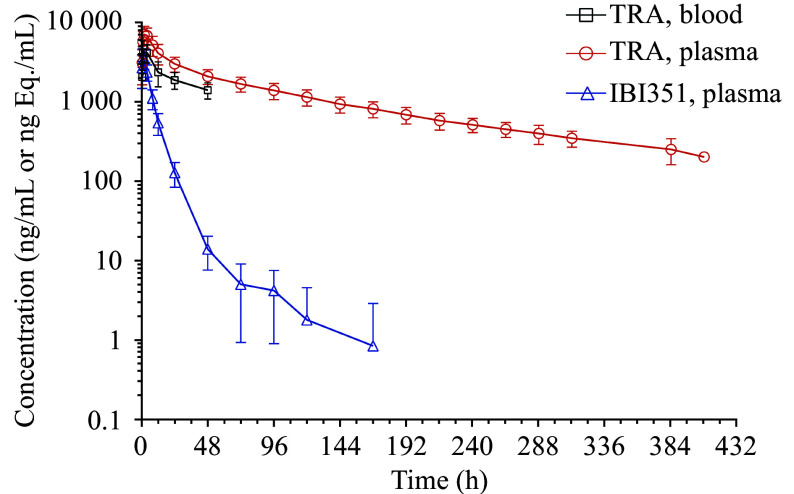
Mean blood drug concentration-time semi-logarithmic curves. Total radioactivity (TRA) determined by liquid scintillation counting in whole blood and plasma (ng Eq./mL) and IBI351 parent drug determined by HPLC-MS/MS in the plasma (ng/mL) after a single oral dose of approximately 600 mg/150 μCi of [^14^C]IBI351. Data are represented as the mean ± standard deviation (*n* = 6).

The TRA in the plasma and the main pharmacokinetic parameters of IBI351 parent drug following a single oral dose of [^14^C]IBI351 in healthy Chinese male subjects are shown in ***Table 1***. The mean blood concentration-time semilog curves for TRA in the whole blood and plasma and IBI351 parent drug in the plasma are shown in ***Fig. 3***. The median *T*_max_ of IBI351 in the plasma was 1.00 h, and the mean *C*_max_ was 3.90 × 10^3^ (± 802) ng/mL. Additionally, the estimated mean *T*_1/2_ was 17.4 (± 11.9) h, the mean *V*_z_/*F* was 581 (± 323) L, and the mean *CL*/*F* was 25.6 (± 5.56) L/h. These results indicate that IBI351 elicits pharmacological effects rapidly and that these effects may endure for a long time. Additionally, IBI351 was widely distributed in the body.

**Table 1:** Major pharmacokinetic parameters of total radioactivity and IBI351 in the plasma of the six healthy Chinese male subjects

Parameters	Total plasma radioactivity	IBI351^b^
*T*_max_^a^ (h)	2.00 (2.00, 4.00)	1.00 (1.00, 2.00)
*C*_max_ (ng Eq./mL)	7.26×10^3^±1.68×10^3^	3.90×10^3^±8.02×10^2^
AUC_0–last_ (h·ng Eq./mL)	4.33×10^5^±1.04×10^5^	2.65×10^4^±5.90×10^3^
AUC_0–inf_ (h·ng Eq./mL)	4.69×10^5^±1.10×10^5^	2.40×10^4^±5.25×10^3^
MRT_0–last_ (h)	108±4.20	8.24±1.10
*T*_1/2_ (h)	129±21.7	17.4±11.9
*V*_z_/*F* (L)	242±50.7	581±323
*CL*/*F* (L/h)	1.32±0.319	25.6±5.56
^a^Median values (min and max) are reported in the table.^b^*C*_max_ and AUC for both parent drug and metabolites are in ng Eq./mL and h·ng Eq./mL, respectively. Abbreviations: *C*_max_, maximum concentration; *T*_max_, time to reach *C*_max_; AUC_0–last_, area under the curve from time zero to the last quantifiable concentration; AUC_0–inf_, area under the curve from time zero to infinity; MRT_0–last_, mean residence time from time zero to the last quantifiable concentration; *T*_1/2_, elimination half-life; *V*_z_/*F*, apparent distribution volume corrected by bioavailability; *CL*/*F*, apparent clearance rate corrected by bioavailability.		

The median *T*_max_ for total plasma radioactivity was 2.00 h, slightly later than the peak time of the IBI351 parent drug. The *C*_max_ was 7.26 × 10^3^ (± 1.68 × 10^3^) ng Eq./mL (***Table 1***). These results suggest that IBI351 is metabolized *in vivo* and may produce high abundances of metabolites, and that the metabolites may have higher protein binding rates than the parent drug. The estimated mean *T*_1/2_ of total radioactive substance in the plasma was 129 (± 21.7) h, indicating that the elimination rate of some metabolites is slower than that of the parent drug.

#### Blood-to-plasma ratio

The radioactive content in whole blood samples from 0 h to 48 h was measured after a single oral dose of 600 mg/150 μCi [^14^C]IBI351. Radioactivity was detected in the whole blood samples from 0.5 h to 48 h. The ratios of total radioactive content in the whole blood to the plasma are presented in ***Table 2***. These ratios were less than 1 and ranged from 0.571 to 0.716, suggesting that [^14^C]IBI351 and related substances did not bind to blood cells on a large scale.

**Table 2:** Ratio of whole blood to total plasma radioactivity after dosing in six healthy Chinese male subjects

Time (h)	Subjects						Mean±SD
	01101	01102	01103	01104	01105	01106	
0	NA	NA	NA	NA	NA	NA	NA
0.50	0.675	0.684	0.768	0.681	0.524	0.596	0.655±0.0841
1.00	0.784	0.722	0.763	0.732	0.632	0.660	0.716±0.0588
4.00	0.614	0.640	0.634	0.598	0.550	0.601	0.606±0.0323
12.0	0.504	0.444	0.650	0.646	0.536	0.644	0.571±0.0884
24.0	0.609	0.646	0.681	0.643	0.564	0.611	0.626±0.0402
48.0	0.681	0.700	0.697	0.683	0.631	0.629	0.670±0.0320
The ratio of total radioactivity in whole blood to the plasma was calculated by dividing the concentration of radioactivity in whole blood (DPM/g) by the concentration of radioactivity in the plasma (DPM/g).Abbreviations: SD, standard deviation; NA, not applicable. The radioactivity intensity in whole blood or the plasma at this time point is below the quantification limit, and thus is not included in the calculation of the mean and standard deviation.							

### Metabolites of IBI351

#### Metabolites identification

**Figure 4:**
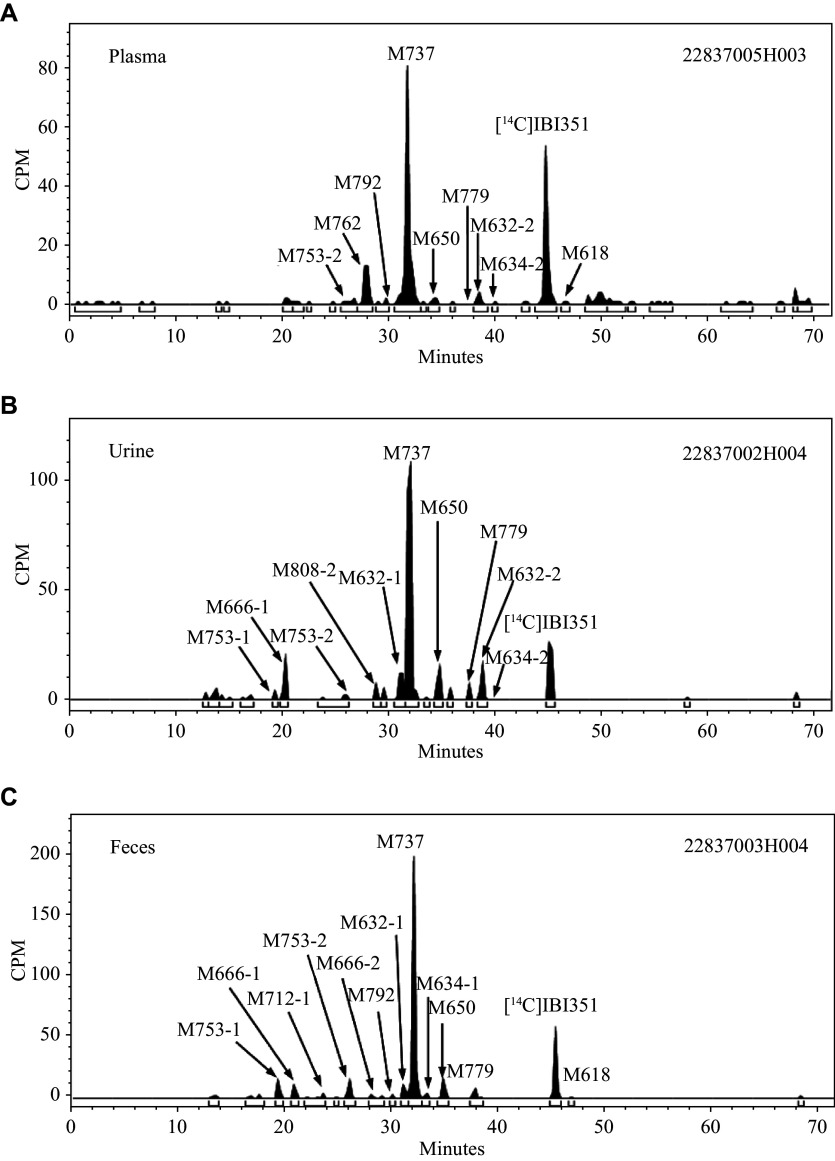
Representative radiochromatograms of IBI351 and metabolites in pooled plasma, urine, and feces. A: The radioactive liquid chromatography profiles of plasma samples from six healthy Chinese male subjects at 0–144 h. B: The radioactive liquid chromatography profiles of urine samples from 0–240 h. C: The radioactive liquid chromatography profiles of fecal samples from 0–192 h. Abbreviation: CPM, counts per minute.

A total of 17 metabolites were identified in the plasma, urine, and fecal samples, including mono-oxidation (M632-1 and M632-2), hydrogenation (M618), dihydrodiol metabolites (M650), mono-oxidation and hydrogenation (M634-1 and M634-2), mono-oxidation and dihydrodiol metabolites (M666-1 and M666-2), glucuronidation (M792), cysteine conjugation (M737), mercapturic acid (M779), and lysine conjugation (M762), as well as mono-oxidation and sulfonation (M712-1 and M712-2), mono-oxidation and glucuronidation (M808-2), and mono-oxidation and cysteine conjugation (M753-1 and M753-2). The mass spectrometry fragments and the percentage of total plasma radioactivity exposure (% of AUC) of the parent drug and its metabolites, as well as the percentage of dose administered (% of dose) in the urine and feces of healthy male subjects, are shown in ***Table 3***. Mass spectrograms of metabolites in the plasma (0–144 h), urine (0–96 h), and feces (0–192 h) are shown in ***Fig. 4A***–***4C***, respectively.

**Table 3:** The levels of [^14^C]IBI351 and its metabolites in total plasma radioactive exposure (% of AUC) and in the urine and feces as a percentage of dose (% of dose)

Parent drug/ metabolites	Metabolite type	[M+H]^+^ (m/z)	Major fragmentation (m/z)	Plasma (0–144 h)			Urine (0–96 h)		Feces (0–192 h)		Feces+urine
				% of AUC (17.74%)	Metabolite/ parent drug (%)^a^		% of dose (9.27%)^b^		% of dose (86.30%)^b^		% of dose (95.56%)^b^
IBI351	Parent drug	617.21	617.21, 484.12, 430.11, 402.11, 358.06, 134.10, 506.14, 112.08, 98.06, 55.02	4.54	100.00		1.16		9.71		10.87
M753-1	Mono-oxidation, cysteine conjugation	754.22	754.22, 667.19, 579.19, 446.10, 418.11, 374.06, 633.20, 112.08, 134.10, 522.13	ND	ND		0.09		1.67		1.76
M753-2				0.17	3.74		0.03		2.94		2.97
M666-1	Mono-oxidation, dihydrodiol	667.21	667.21, 579.19, 446.10, 418.11, 374.06, 134.10, 522.13, 146.08, 86.06	ND	ND		0.01		1.76		1.77
M666-2				ND	ND		ND		0.97		0.97
M712-1	Mono-oxidation, sulfonation	713.16	713.16, 633.20, 500.11, 446.10, 418.11, 374.06, 134.10, 522.13, 112.08, 98.06, 55.02	ND	ND		ND		0.52		0.52
M712-2				ND	ND		ND		0.07		0.07
M762	Lysine conjugation	763.31	763.31, 563.20, 430.11, 402.11, 358.06, 605.21, 134.10, 506.14	1.58	34.80		0.18		ND		0.18
M808-2	Mono-oxidation, glucuronidation	809.23	809.23, 633.20, 500.11, 446.10, 418.11, 374.06, 134.10, 522.13, 112.078, 98.06	ND	ND		0.04		ND		0.04
M792	Glucuronidation	793.24	793.24, 617.21, 112.08, 484.12, 430.11, 402.11, 358.06, 134.10, 506.14	0.11	2.42		ND		0.61		0.61
M632-1	Mono-oxidation	633.20	633.20, 500.11, 446.10, 418.11, 374.06, 134.10, 522.13, 112.08, 98.06, 55.02	ND	ND		0.86		1.79		2.65
M632-2				0.32	7.05		0.39		0.43		0.82
M737	Cysteine conjugation	738.23	738.23, 651.20, 563.20, 430.11, 402.11, 358.06, 617.21, 134.10, 506.14, 112.08	6.17	135.90		5.49		39.87		45.36
M634-1	Mono-oxidation, hydrogenation	635.22	635.22, 502.13, 446.10, 418.11, 374.06, 134.10, 522.13, 114.09, 100.08	ND	ND		ND		1.36		1.36
M634-2				0.08	1.76		0.03		0.27		0.30
M650	Dihydrodiol	651.21	651.21, 563.20, 430.11, 402.11, 358.06, 134.10, 506.14, 146.08, 86.06	0.27	5.95		0.23		2.47		2.70
M779	Mercapturic acid	780.24	780.24, 651.20, 563.20, 430.11, 402.11, 358.06, 617.21, 134.10, 506.14, 112.08	0.05	1.10		0.11		2.59		2.70
M618	Hydrogenation	619.22	619.22, 486.13, 430.11, 402.11, 358.06, 134.10, 506.14, 100.08	0.09	1.98		ND		0.35		0.35
PES				82.26			NA		16.72		16.72
Total identification peaks^c^				13.38			8.62		67.38		76.00
Total unidentified peaks^d^				4.36			0.65		2.20		2.85
Percentage of peaks identified^e^				95.64			92.99		97.45		97.03
^a^The percentage of metabolites relative to the parent drug in the plasma.^b^The percentage of urine and feces excretion from 0 to 360 h relative to the administered dose.^c^The percentage of identified peaks in the plasma relative to the total radioactivity exposure or dose administered.^d^Individual radioactive peaks do not exceed 1.00% of the AUC, or 1.00% of the dose.^e^The sum of identified peaks as a percentage of the current matrix.There may be an error of ±0.1 due to rounding.Abbreviations: AUC, area under the concentration-time curve; ND, not detected; PES: post-extracted solid residue.											

#### Circulating radioactivity

The extraction rate in the plasma samples decreased over time, until it reached 13.18% at 144 h. The extractable fraction in the plasma, including the parent drug, M737, M762, M753-2, M792, M650, M779, M632-2, M634-2, and M618, fell below the detection limit (50 DPM/g) after 168 h. The unextractable fraction in the plasma accounted for 82.26% of the total radioactive exposure in the plasma (AUC_0–last_), suggesting that this fraction was the predominant radioactive species in the plasma and was a covalent conjugate formed specifically with the plasma albumin. The parent drug was the major radioactive component in the extractable fraction, accounting for 4.54% of the TRA exposure. The main metabolite in the plasma was M737, accounting for 6.17% of the TRA exposure. The minor metabolite was M762, accounting for 1.58% of the TRA exposure. Other metabolites M753-2, M792, M650, M779, M632-2, M634-2, and M618 each accounted for 0.05% to 0.32% of the TRA exposure. The unidentified radioactive areas did not exceed 0.99% of the TRA exposure in the plasma.

#### Metabolites in the feces

After the administration of a single oral dose of [^14^C]IBI351, the drug was primarily excreted through the feces. The TRA excreted into the feces from 0 h to 192 h accounted for 86.30% of the administered dose. The unextractable fraction in the feces comprised covalent conjugates formed between the parent drug-related substances and fecal matrix, accounting for approximately 16.72% of the administered dose, and the parent drug accounted for 9.71%. The main metabolite in the feces was M737, accounting for 39.87%. The minor metabolites were M753-1, M666-1, M753-2, M632-1, M634-1, M650, and M779, accounting for 1.67%, 1.76%, 2.94%, 1.79%, 1.36%, 2.47%, and 2.59%, respectively. Other metabolites, including M712-1, M666-2, M792, M712-2, M632-2, M634-2, and M618, each accounted for 0.07% to 0.97%. The unidentified radioactive areas were not higher than 0.80% of the administered dose.

#### Metabolites in the urine

The TRA excreted into the urine from 0 h to 96 h accounted for 9.27% of the administered dose, with identified radioactivity peaks accounting for 8.62%, and the parent drug accounted for only 1.16%. The main metabolite M737 accounted for 5.49%. Other metabolites, including M753-1, M666-1, M753-2, M762, M808-2, M632-1, M650, M779, M632-2, and M634-2, each accounted for 0.01% to 0.86%. The unidentified radioactive areas were not higher than 0.25% of the administered dose.

#### Metabolic pathways

**Figure 5:**
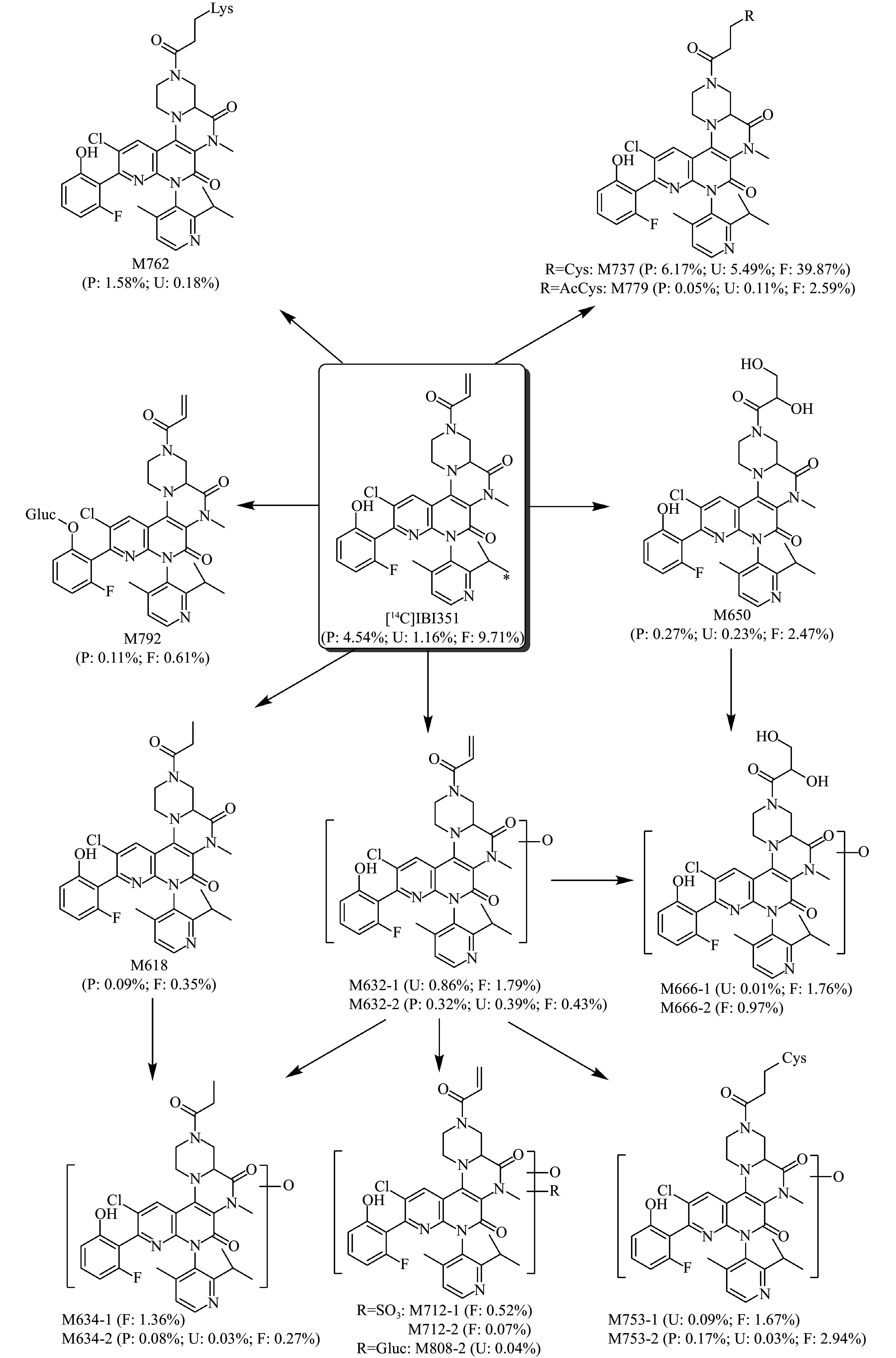
Possible metabolic pathways of IBI351 in humans. Asterisk (*) indicates the position of ^14^C-labeling; P represents plasma, expressed as % of AUC; U and F represent urine and feces, expressed as % of dose. Abbreviations: AUC, area under the concentration-time curve; Lys, lysine; AcCys, N-acetyl-cysteine; SO_3_, sulfonate; Gluc, glucose; Cys, cysteine.

According to the metabolites of IBI351, the main metabolic pathways might involve (1) oxidation, (2) hydrogenation, (3) sulfonate conjugation, (4) glucuronide conjugation, and (5) cysteine conjugation (***Fig. 5***).

### Safety

The vital signs (*i.e.*, body temperature, pulse, respiratory rate, and blood pressure), physical indexes, and electrocardiogram results of the six subjects were within normal range before and after the administration, suggesting that the present IBI351 doses were generally safe and well tolerated (***Supplementary Table 4*** [available online]). The six subjects completed this clinical trial, with four subjects (66.7%) experiencing treatment-emergent adverse events (TEAEs) unrelated to [^14^C]IBI351, including elevated blood triglycerides (33.3%), diarrhea (33.3%), and positive fecal occult blood (16.7%), all of which were classified as grade 1 severity. No interventions were performed for any adverse events, and all the subjects eventually recovered. There were no TEAEs with Common Terminology Criteria for Adverse Events (CTCAE) grade ≥ 3, no serious adverse events, and no TEAEs leading to death.

## Discussion

The clinical study evaluated the absorption, metabolism, and excretion of a single oral dose of [^14^C]IBI351 in healthy Chinese male subjects. Following administration of 600 mg/150 µCi, the median *T*_max_ was 1 h for the parental drug IB1351 and 2 h for TRA in the plasma, indicating that the metabolites appear slightly later. Their *C*_max_ values, expressed as equimolar concentrations, were 3.90 × 10^3^ (± 8.02 × 10^2^) ng Eq./mL and 7.26 × 10^3^ (± 1.68 × 10^3^) ng Eq./mL, respectively, with a subsequent decrease in concentration following the peak. The quantifiable time points were 168 h and 456 h, respectively, and the estimated *T*_1/2_ values were 17.4 h and 129 h, respectively. The half-life of the TRA was longer than that of the parent drug, which is similar to the findings in mass balance studies of other specific covalent small molecule inhibitors targeting the same pathway, such as osimertinib and sotorasib^[17–18]^. This may be due to the covalent binding of the drug to plasma proteins. Additionally, we conducted a covalent binding analysis of IBI351 in the plasma, and the results indicated the presence of covalent binding between IBI351 and albumin.

The TRA of metabolites excreted into the urine and feces accounted for 95.56% (± 3.41%) of the administered dose (0–192 h). Fecal excretion accounted for approximately 86.30% (± 4.90%), while renal excretion accounted for 9.27% (± 1.83%), indicating that renal clearance was not the main elimination pathway for IBI351. The main metabolite in the feces was cysteine conjugate M737, accounting for 39.87%.

In the current study, we identified and quantified the metabolites of IBI351. The metabolite M737 accounted for 6.17% of the TRA exposure in the plasma, while M762 accounted for 1.58%. Other metabolites, including M753-2, M792, M650, M779, M632-2, M634-2, and M618, each accounted for 0.05% to 0.32%. Since the structures of these metabolites are similar to the structure of IBI351, the metabolites may also induce inhibition of the covalent activity of KRAS. Nevertheless, the pharmacological effects of IBI351 metabolites require further experimental validation in the future.

According to the metabolites of IBI351, we hypothesized that the main metabolic pathways of IBI351 in healthy male subjects include (1) oxidation, (2) hydrogenation, (3) sulfonate conjugation, (4) glucuronide conjugation, and (5) cysteine conjugation. A major metabolic enzyme is defined as an enzyme that contributes to at least 25% of the elimination of a drug. IBI351 did not show any significant metabolic enzyme in its elimination, which may reduce the risk of drug-drug interactions and pharmacogenetic variability.

In the current study, all TEAEs observed were of grade 1 and not related to [^14^C]IBI351. There were no TEAEs of CTCAE grade ≥ 3, no severe adverse events, and no TEAEs leading to death. Therefore, the subjects showed a good safety and tolerance to a single oral dose of [^14^C]IBI351 of 600 mg/150 µCi.

In the current study, we compared the experimental results of IBI351 with those of two commercially available KRAS covalent inhibitors, sotorasib and adagrasib^[11,19]^. The average time to reach *C*_max_ for sotorasib and adagrasib was 1 h and 6 h, respectively, whereas it was 1 h for IBI351, indicating that IBI351, like sotorasib, acts more rapidly than adagrasib. Additionally, sotorasib has a terminal elimination half-life of 5 h, while that of adagrasib is 23 h. IBI351, with a half-life of 17.4 h, showed a longer duration of pharmacological effect compared with sotorasib. Moreover, compared with sotorasib, which has a volume of distribution of 211 L and a plasma protein binding rate of 89%, and adagrasib, which has a higher volume of distribution of 942 L and a plasma protein binding rate of 98%, IBI351 exhibited an apparent volume of distribution of 581 L and a plasma protein binding rate of 82.26%. This indicates that IBI351 has a wider distribution in the body than sotorasib and a shorter time to peak than adagrasib, thus exerting pharmacological effects more quickly. Furthermore, sotorasib is primarily metabolized through non-enzymatic conjugation and oxidative processes involving CYP3A enzymes, while adagrasib is primarily metabolized by CYP3A4. Notably, IBI351 did not show significant involvement of major metabolic enzymes in its elimination, suggesting a lower risk of drug interaction, whereas sotorasib and adagrasib are often challenged with significant adverse events related to drug-drug interactions. Importantly, all three drugs are primarily excreted *via* feces. As for safety, both sotorasib and adagrasib are associated with grade ≥ 3 adverse events, whereas all adverse events from IBI351 were of grade 1, suggesting that IBI351 may have a better safety. Overall, IBI351 presents pharmacokinetic characteristics much more similar to those of sotorasib, and may be safer than the present KRAS covalent inhibitors.

In summary, we reported for the first time the mass balance, metabolism, excretion, and safety of IBI351 in healthy Chinese subjects. The mean cumulative excreted radioactivity reached 95.56% at 312 h, indicating that the excretion of radioactivity in humans is almost complete. The main metabolic pathways of IBI351 in humans include oxidation, hydrogenation, sulfonate conjugation, glucuronide conjugation, and cysteine conjugation. Fecal excretion was the main route of the elimination of IBI351. Moreover, IBI351 showed a better safety profile at a dose of 600 mg/150 µCi. Nevertheless, the sample size should be further expanded to validate the safety and efficacy of IBI351 in future clinical trials. Notably, due to the use of radiolabeled drugs in the current study, female subjects were not included because of concerns for fertility safety. Additional experimental evidence is needed to confirm the pharmacokinetic characteristics of IBI351 in female subjects.

## SUPPLEMENTARY DATA

Supplementary data to this article can be found online.
