# Supplementary material for: Study of the mass balance, biotransformation, and safety of [14C]IBI351 in healthy Chinese subjects
Source: J Biomed Res. 2024 Oct 22;39(4):382–93. doi: 10.7555/JBR.38.20240254 (PMC12329412; doi:10.7555/JBR.38.20240254)
Supplement: Supplementary file 1 — Supplementary data to this article can be found online. [file jbr-39-4-382-Supplementary.pdf]

Available online at www.jbr-pub.org.cn

Open Access at PubMed Central

JBR

Journal of Biomedical Research, 2025 39(4): 1–3

Supplementary Data

Study of the mass balance, biotransformation, and safety of [^{14}C]IBI351 in healthy Chinese subjects

Shuaishuai Wang[1,△], Wen Lin[1,△], Bilal Ahmed[1], Tianqi Zhong[2], Jun Zhao[2], Lijun Xie[2], Hao Feng[3], Juan Chen[2], Chen Zhang[4], Peng Yan[4], Shirui Zheng[5], Lingge Cheng[5], Yipeng Cheng[5], Bei Zhu[2], Feng Han[1,✉], Lulu Zhang[1,✉], Chen Zhou[2,✉]

[1]*Medical Basic Research Innovation Center for Cardiovascular and Cerebrovascular Diseases, Ministry of Education, International Joint Laboratory for Drug Target of Critical Illnesses, Key Laboratory of Cardiovascular and Cerebrovascular Medicine, School of Pharmacy, Nanjing Medical University, Nanjing, Jiangsu 211166, China;*
[2]*Phase Ⅰ Clinical Trial Unit, the First Affiliated Hospital of Nanjing Medical University, Nanjing, Jiangsu 210029, China;*
[3]*Value Pharmaceutical Services Co., Ltd., Nanjing, Jiangsu 211806, China;*
[4]*Nuclear Medicine Department, the First Affiliated Hospital of Nanjing Medical University, Nanjing, Jiangsu 210029, China;*
[5]*Innovent Biologics Co., Ltd., Shanghai 201107, China.*

Supplementary Table 1 **Demographic characteristics and baseline features of the six healthy Chinese male subjects**

Variables	Value
Age (years)[a]	
Mean (SD)	30 (5)
Median (range)	30 (23–36)
Male [*n* (%)]	6 (100)
Race [*n* (%)]	
Asian	6 (100)
Others	0
Ethnicity, *n* (%)	
Han Chinese	6 (100)
Others	0

△These authors contributed equally to this work.

✉Corresponding authors: Chen Zhou, Phase Ⅰ Clinical Trial Unit, the First Affiliated Hospital of Nanjing Medical University, 300 Guangzhou Road, Nanjing, Jiangsu 210029, China. E-mail: jsphkjzc@163.com; Lulu Zhang and Fen Han, Medical Basic Research Innovation Center for Cardiovascular and Cerebrovascular Diseases, Ministry of Education International Joint Laboratory for Drug Target of Critical Illnesses, Key Laboratory of Cardiovascular and Cerebrovascular Medicine, School of Pharmacy, Nanjing Medical University, 101 Longmian Avenue, Nanjing, Jiangsu 211166, China. E-mails: lulu_0219@njmu.edu.cn (Zhang) and fenghan169@njmu.edu.cn (Han).

Received: 26 August 2024; Revised: 19 September 2024; Accepted: 20 September 2024; Published online: 22 October 2024

CLC number: R965, Document code: A

Author Hao Feng was employed by the company Value Pharmaceutical Services Co., Ltd. Authors Shirui Zheng, Lingge Cheng, and Yipeng Cheng were employed by the company Innovent Biologics Co., Ltd., Shanghai, China. The remaining authors authors reported no conflict of interests.

This is an open access article under the Creative Commons Attribution (CC BY 4.0) license, which permits others to distribute, remix, adapt and build upon this work, for commercial use, provided the original work is properly cited.

© 2025 by Journal of Biomedical Research.

https://doi.org/10.7555/JBR.38.20240254

Supplementary Table 1 **Demographic characteristics and baseline features of the six healthy Chinese male subjects** (Continued)

Variables	Value
Height (cm)	
Mean (SD)	174 (4)
Median (range)	174 (169–182)
Weight (kg)	
Mean (SD)	70 (6)
Median (range)	72 (60–75)
Body mass index (kg/m^2)	
Mean (SD)	23 (1)
Median (range)	23 (21–25)
Drinking history, *n* (%)	
Never	6 (100)
Smoking history, *n* (%)	
Never	6 (100)
Drug abuse screening, *n* (%)	
Negative	6 (100)

[a]Age (years) = (informed consent date − date of birth + 1)/365.25, rounded down.
Abbreviation: SD, standard deviation.

Supplementary Table 2 **The actual doses administered to six healthy subjects**

Variables	Subject						Mean±SD
	01101	01102	01103	01104	01105	01106	
IBI351 (mg)	590	586	592	591	592	592	591±2.35
Radioactivity (μCi)	145	144	146	146	146	146	146±0.837

Abbreviation: SD, standard deviation.

Supplementary Table 3 **Recovered radioactivity in excreta after a single oral dose**

Variables	Subject						Mean±SD
	01101	01102	01103	01104	01105[a]	01106	
Urine (% of dose)	11.69	7.48	11.16	8.65	7.33	9.26	9.27±1.83
Feces (% of dose)	80.67	86.99	80.29	87.83	92.67	89.35	86.30±4.90
Urine and feces (% of dose)	92.36	94.47	91.45	96.48	100.00	98.61	95.56±3.41

[a]Due to the total radioactive recovery in urine and feces exceeding 100.00% in the subject 01105, the data were normalized to a recovery rate of 100.00%.
Abbreviation: SD, standard deviation.

Supplementary Table 4 **Safety data of six healthy subjects**

Subject	Item	Reference range	The administration of [^{14}C]IBI351			Follow-up visit
			Before	D3	D8	
01101	ALT (U/L)	9.0–50.0	9.5	11.4	18.3	9.5
	AST (U/L)	15.0–40.0	19.1	18.5	24.1	15.7
	GGT (U/L)	10.0–60.0	12.9	9.9	12.1	13.4
	TBIL (μmol/L)	5.1–19.0	12.1	6.4	8.1	8.1
	Urea (mmol/L)	3.1–8.0	3.9	4.3	3.8	3.5
	Cr (μmol/L)	57.0–97.0	70.4	73.1	69.6	72.8

Supplementary Table 4 **Safety data of six healthy subjects** (Continued)

Subject	Item	Reference range	The administration of [^{14}C]IBI351			Follow-up visit
			Before	D3	D8	
01102	ALT	9.0–50.0	9.9	10.3	12.1	8.0
	AST	15.0–40.0	14.8	10.9	15.2	11.0
	GGT	10.0–60.0	17.3	14.4	17.0	15.9
	TBIL	5.1–19.0	15.4	12.2	14.2	12.3
	Urea	3.1–8.0	6.2	4.9	5.4	3.8
	Cr	57.0–97.0	78.2	69.5	73.7	73.1
01103	ALT	9.0–50.0	12.6	11.0	10.5	20.5
	AST	15.0–40.0	15.6	11.5	14.2	14.2
	GGT	10.0–60.0	22.0	17.1	25.8	25.8
	TBIL	5.1–19.0	12.4	7.7	4.5	4.5
	Urea	3.1–8.0	5.4	5.1	5.6	5.6
	Cr	57.0–97.0	73.5	66.4	64.2	64.2
01104	ALT	9.0–50.0	16.6	14.4	19.8	19.4
	AST	15.0–40.0	16.6	16.4	15.8	16.9
	GGT	10.0–60.0	20.8	19.1	16.8	22.0
	TBIL	5.1–19.0	8.0	10.0	8.0	7.0
	Urea	3.1–8.0	4.5	4.5	4.2	4.7
	Cr	57.0–97.0	68.6	69.2	72.0	71.4
01105	ALT	9.0–50.0	22.8	29.0	30.9	21.4
	AST	15.0–40.0	24.6	23.6	22.2	19.4
	GGT	10.0–60.0	32.0	33.0	33.8	32.9
	TBIL	5.1–19.0	12.9	11.5	15.1	7.0
	Urea	3.1–8.0	3.3	4.0	4.5	4.1
	Cr	57.0–97.0	74.1	76.5	76.4	66.8
01106	ALT	9.0–50.0	17.2	17.0	17.9	14.7
	AST	15.0–40.0	21.1	19.0	17.7	17.6
	GGT	10.0–60.0	19.9	20.1	19.2	16.9
	TBIL	5.1–19.0	18.1	10.4	13.5	8.8
	Urea	3.1–8.0	4.1	5.5	5.1	6.1
	Cr	57.0–97.0	82.4	78.7	84.3	92.4

Abbreviations: ALT, alanine aminotransferase; AST, aspartate aminotransferase; GGT, L-γ-glutamyl transpeptidase; TBIL, total bilirubin; Cr, creatinine.
